# Cardiac Tamponade Associated with the Presentation of Anaplastic Large Cell Lymphoma in a 2-Year-Old Child

**DOI:** 10.1155/2015/487491

**Published:** 2015-09-08

**Authors:** Gema Mira-Perceval Juan, Pedro J. Alcalá Minagorre, Ana M. Huertas Sánchez, Sheila Segura Sánchez, Silvia López Iniesta, Francisco J. De León Marrero, Estela Costa Navarro, María Niveiro de Jaime

**Affiliations:** ^1^Department of Pediatrics, University General Hospital of Alicante, C/Pintor Baeza 12, 03010 Alicante, Spain; ^2^Department of Pediatric Hematology and Oncology, University General Hospital of Alicante, C/Pintor Baeza 12, 03010 Alicante, Spain; ^3^Department of Dermatology, University General Hospital of Alicante, C/Pintor Baeza 12, 03010 Alicante, Spain; ^4^Department of Pathological Anatomy, University General Hospital of Alicante, C/Pintor Baeza 12, 03010 Alicante, Spain

## Abstract

The anaplastic large cell lymphoma is a rare entity in pediatric patients. We present an unusual case of pericardial involvement, quite uncommon as extranodal presentation of this type of disorder, that provoked a life-risk situation requiring an urgent pericardiocentesis. To our knowledge, this is the first report on a child with pericardial involvement without an associated cardiac mass secondary to anaplastic large cell lymphoma in pediatric age. We report the case of a 21-month-old Caucasian male infant with cardiac tamponade associated with the presentation of anaplastic large cell lymphoma. Initially, the child presented with 24-day prolonged fever syndrome, cutaneous lesions associated with hepatomegaly, inguinal adenopathies, and pneumonia. After a 21-day asymptomatic period, polypnea and tachycardia were detected in a clinical check-up. Chest X-ray revealed a remarkable increase of the cardiothoracic index. The anaplastic large cell lymphoma has a high incidence of extranodal involvement but myocardial or pericardial involvements are rare. For this reason, we recommend a close monitoring of patients with a differential diagnosis of anaplastic large cell lymphoma.

## 1. Introduction

The anaplastic large cell lymphoma (ALCL) is a rare entity in pediatric patients. It represents approximately 10% of lymphomas in children [1] and it is included in the non-Hodgkin lymphomas group according to the 2008 WHO classification [2]. Two entities are identified according to clinical and molecular criteria, the primary cutaneous anaplastic large cell lymphoma (c-ALCL) and the primary systemic anaplastic large cell lymphoma (s-ALCL), which is divided into 2 subtypes according to the expression of the anaplastic lymphoma kinase (ALK) [2].

Up to 70% of the pediatric patients with s-ALCL present with a disseminated stage of the disease at diagnosis [3]. Constitutional symptoms and signs appear quite often: fever, asthenia, anorexia, and weight loss. This lymphoma has a high incidence of extranodal involvement and it is the pediatric lymphoma with the highest skin affinity. The central nervous system (CNS), lungs, bone marrow, spleen, and liver may also be involved. Cases of ALCL with myocardial [4] or pericardial [5] involvement are rare. To the best of our knowledge, this is the first report on a child with pericardial involvement without an associated cardiac mass secondary to ALCL in pediatric age.

## 2. Case Presentation

**Figure 1:**
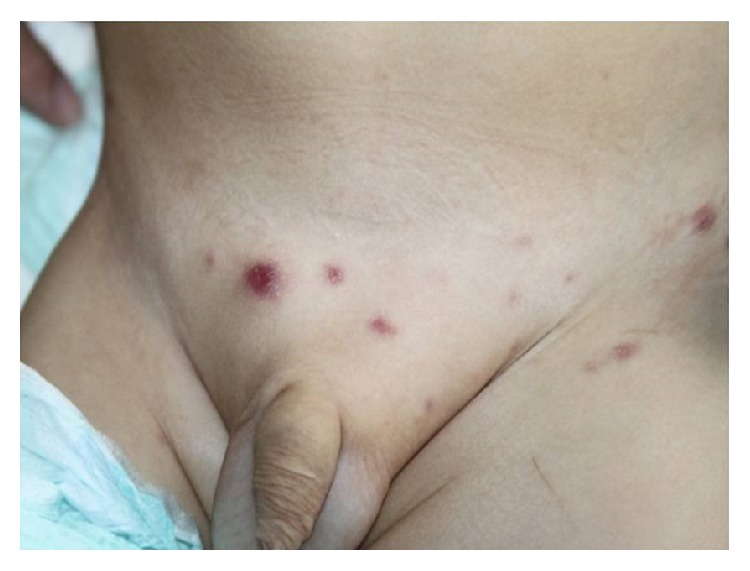
Papulosquamous lesions located in the inguinal region.

A 21-month-old baby with a 24-day prolonged fever syndrome, cutaneous lesions associated with hepatomegaly, inguinal adenopathies, and pneumonia located in the middle lobe with a small pleural effusion was admitted to our centre. Cutaneous lesions were papulosquamous and located in the inguinal region and trunk (Figure 1).

Initially, amoxicillin-clavulanate therapy was administered. An expectant attitude was adopted before the pleural effusion due to its small size. The patient progressed favorably in the short run; he was afebrile within 24 hours; respiratory involvement was less severe; in the following days, cutaneous lesions progressively disappeared and adenopathies were reduced.

Complete blood count, blood culture, and the tuberculin skin test did not show pathologic results. Peripheral blood studies did not report atypical cells. The serum test showed a weak positive IgM for* Chlamydia pneumoniae.* However, due to the cutaneous lesions and the history of prolonged febrile syndrome, a lymphoproliferative disorder was considered as possible diagnosis. A biopsy of the cutaneous lesions was performed for anatomopathological and immunohistochemical analysis. Chest X-ray showed disappearance of the lobe consolidation and the pleural effusion 2 weeks after admission. Before the clinical stability, the patient was discharged and followed ambulatory care.

**Figure 2:**
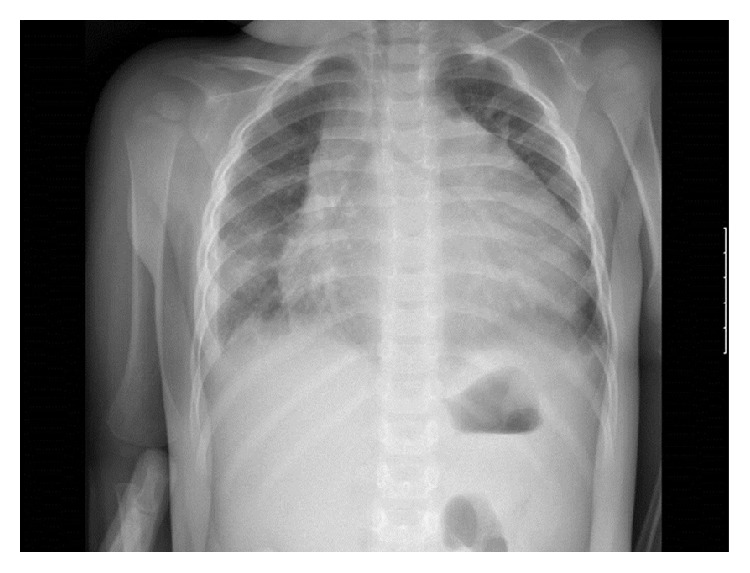
Chest X-ray. Increase of the cardiothoracic index at the time of diagnosis of cardiac tamponade. The patient had a normal chest X-ray 10 days before.

After a 21-day asymptomatic period, polypnea and tachycardia were detected in a clinical check-up. Chest X-ray revealed a remarkable increase of the cardiothoracic index (Figure 2). The echocardiographic study showed a massive pericardial effusion causing cardiac tamponade. A pericardiocentesis was performed to drain 180 mL of serohematic fluid.

**Figure 3:**
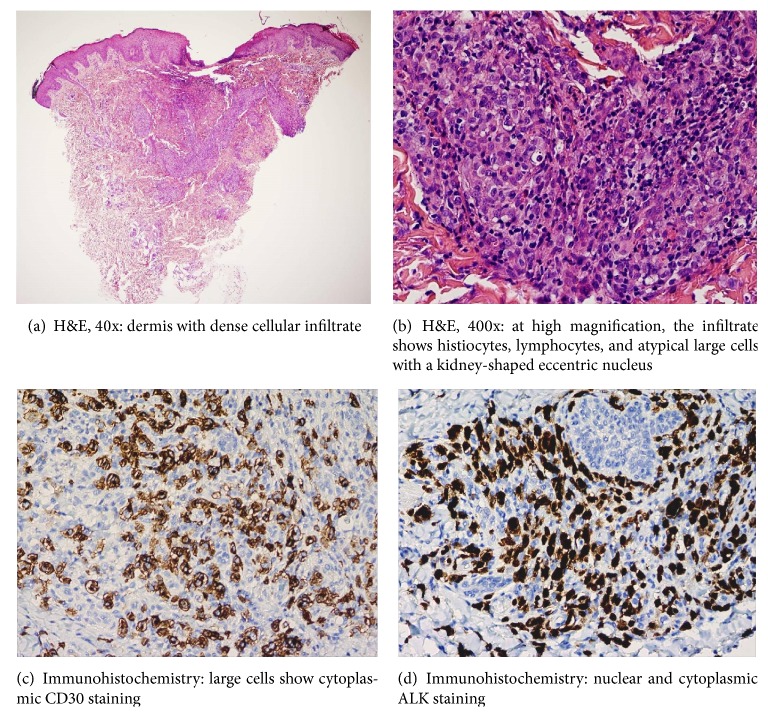
Skin biopsy.

The pericardial fluid analysis showed infiltration due to anaplastic lymphoma CD30+. Cutaneous biopsy revealed a CD30+, ALK+ lymphoproliferative process (Figure 3). Cutaneous and pericardial involvement in addition to the prolonged fever history and the likely respiratory involvement led to the diagnosis of anaplastic lymphoma s-ALCL, ALK+ stage IV-B. Assessment of tumor extension (CT scan, bone marrow aspiration, abdominal ultrasound, pulmonary CT angiography, head MRI, and CSF analysis) did not show other affected organs.

Chemotherapy treatment was administered according to ALCL international protocol (2003) high risk subgroup. It consisted of an initial cytoreductive phase followed by 6 cycles (AM/BM) administered alternately every 21 days.

Twelve months after completion of therapy, the patient has progressed favorably without evidence of residual disease.

## 3. Discussion

Diagnosis of systemic ALCL is based on morphological (large cells with a kidney-shaped eccentric nucleus), immunohistochemical, and genetic criteria [6]. ALK protein is overexpressed when translocation occurs (2;5) (p23;q35) and has been described in more than 80% of ALCL cases in children. Positive ALK lymphomas have a better prognosis than negative ALK lymphomas [7]. Protein expression is normally found by immunohistochemical techniques in the tumor cells of the CD30 antigen, the epithelial membrane antigen (EMA), and the interleukin-2 receptor (CD25).

Neoplastic cells in c-ALCL rarely express ALK and a spontaneous resolution is produced in up to 40% of cases. Thus, primary c-ALCL may be difficult to distinguish from lymphomatoid papulosis (LyP) clinically and histologically. Both entities are rare in children. Isolated or multiple lesions concentrated on an anatomic region suggest a c-ALCL, whereas the disseminated lesions are more common in the LyP [8]. The difference between c-ALCL and LyP may be difficult in clinical practice, because they are clinically, histologically, and immunohistologically similar and even some authors consider them as spectrum within the same disorder [9]. Moreover, distinguishing between systemic and primary cutaneous ALCL may be complicated [10], especially at an initial stage.

In our case, diagnosis was difficult at an early stage due to the self-limited character of the pulmonary process and the initial clinical improvement. Due to the lack of biological sample, it could not be established whether the lobe consolidation and the effusion were related to the extranodal involvement that occurs in 10% of cases or it was a recurrent infectious process (weak positive IgM for* Chlamydia pneumoniae*). Diagnosis was precipitated by the appearance of cardiac tamponade and the cytologic and immunohistochemical findings of the pericardial fluid together with the final results of the cutaneous biopsy.

Pericardial involvement is quite uncommon as extranodal presentation of s-ALCL, but it may provoke a life-risk situation requiring an urgent pericardiocentesis, as was the case in our patient. For this reason, we recommend a close monitoring of patients with a differential diagnosis of CD30+ lymphoproliferative disorder.

## Abbreviations

ALCL: Anaplastic large cell lymphoma
ALK: Anaplastic lymphoma kinase
CNS: Central nervous system
c-ALCL: Primary cutaneous anaplastic large cell lymphoma
EMA: Epithelial membrane antigen
LyP: Lymphomatoid papulosis
s-ALCL: Systemic anaplastic large cell lymphoma.
